# Anti-Pancreatic Cancer Deliverables from Sea: First-Hand Evidence on the Efficacy, Molecular Targets and Mode of Action for Multifarious Polyphenols from Five Different Brown-Algae

**DOI:** 10.1371/journal.pone.0061977

**Published:** 2013-04-16

**Authors:** Sheeja Aravindan, Caroline R. Delma, Somasundaram S. Thirugnanasambandan, Terence S. Herman, Natarajan Aravindan

**Affiliations:** 1 Department of Radiation Oncology, University of Oklahoma Health Sciences Center, Oklahoma City, Oklahoma, United States of America; 2 Department of Marine Sciences, Center of Advanced Study in Marine Biology, Annamalai University, Parangipettai, India; Wayne State University School of Medicine, United States of America

## Abstract

Pancreatic cancer (PC) remains the fourth leading cause of cancer death with an unacceptable survival that has remained relatively unchanged over the past 25 years. The presence of occult or clinical metastases at the time of diagnosis together with the lack of effective chemotherapies pose a dire need for designing new and targeted therapeutic deliverables that favors the clinical outcome. Herein, we investigated the anti-tumorigenic potential of polyphenols from five different brown-algae in human PC cells (MiaPaCa-2, Panc-1, BXPC-3 and Panc-3.27). Total anti-oxidant capacity (TAC) analysis on stepwise polyphenol separations with increasing polarity (Hexane-DCM-EA-methanol) identified high levels of TAC in DCM and EA extractions across all seaweeds assessed. All DCM and EA separated polyphenols induced a dose-dependent and sustained (time-independent) inhibition of cell proliferation and viability. Further, these polyphenols profoundly enhanced DNA damage (acridine orange/Ethidium bromide staining and DNA fragmentation) in all the cell lines investigated. More importantly, luciferase reporter assay revealed a significant inhibition of NFκB transcription in cells treated with polyphenols. Interestingly, QPCR analysis identified a differential yet definite regulation of pro-tumorigenic EGFR, VEGFA, AKT, hTERT, kRas, Bcl2, FGFα and PDGFα transcription in cells treated with DCM and EA polyphenols. Immunoblotting validates the inhibitory potential of seaweed polyphenols in EGFR phosphorylation, kRas, AurKβ and Stat3. Together, these data suggest that intermediate polarity based fractions of seaweed polyphenols may significantly potentiate tumor cell killing and may serve as potential drug deliverable for PC cure. More Studies dissecting out the active constituents in potent fractions, mechanisms of action and synergism, if any, are warranted and are currently in process.

## Introduction

Pancreatic cancer (PC) remains the fourth leading cause of cancer death in the United States with an expected 43,920 new cases in 2012 [1]. Unfortunately, the PC mortality rates remained relatively unchanged since 1975, with a 5-year survival rate of only 5.4% [2]. The presence of occult or clinical metastases at the time of diagnosis together with the lack of effective chemotherapies contributes to the high mortality in patients with PC. To that end, treatment of PC, highly rely on the improved understanding of genetics and biology [3], which may be closely associated with tumorigenesis and metastasis formation. Also, PC is one of the most intrinsically drug-resistant tumors and, resistance to chemotherapeutic agents is a major cause of treatment failure in PC. Despite extensive research on many targeted therapies with excellent anti-tumor effects in pre-clinical models, clinical investigation showed that single targeted therapies and most combined therapies were not able to improve the prognosis of this disease. It is noteworthy to mention that there are 1344 ongoing clinical trials (www.clinical trials.gov accessed on November 30, 2012) at present, targeting many potential molecular targets with a number of promising pipe-line drug-deliverables. Despite, such a colossal attempt to mitigate PC progression and spreading, in real-time, we are in nowhere near the acceptable survival rates. Partly, this is attributed to the fact that PC may possess different characteristics and targets in different stages of pathogenesis, maintenance and metastasis [4]. Further, cross-talk between cell signaling pathways, an important phenomenon in PC, may result in cancer cell survival and metastasis, even though some pathways are blocked by targeted therapy. Sensitivity to therapy may also vary for cancer cells at different stages. The unique PC structure with abundant stroma creates a tumor microenvironment with hypoxia and low blood perfusion rate, which prevents drug delivery to cancer cells. Therefore there is a calamitous need for designing new and targeted therapeutic strategies that can improve the clinical outcome for patients diagnosed with PC. Accordingly, herein we investigated the anti-PC potential of polyphenols from an unusual source, marine brown algae.

Macro algae (sea weeds) are important sources of protein, iodine, vitamins and minerals and hence have shown to possess chemo preventive potentials [5]. Further studies have consistently demonstrated that, the seaweeds possess a high content of polyphenols such as catechin, epicatechin, epigallocatechin galate and gallic acid (see review [6]). These polyphenolic compounds have shown many health benefiting bioactivities such as anti-oxidant, anti-cancer, anti-viral, anti-inflammatory, and an ability to inhibit human platelet aggregation 6,7. In addition studies have shown a positive correlation between the increased dietary intake of natural antioxidants and the reduced coronary heart disease, cancer mortality, as well as longer life expectancy [6], [8]. A close association between anti-carcinogenic activity and antioxidant activity has been reported in mouse models of carcinoma with polyphenols [9]–[12]. To that note, recent investigations precisely demonstrated the anti-proliferative, pro-apoptotic, DNA damaging, anti-angiogenic, growth inhibiting, cell-cycle arrest and anti-metastatic functions of seaweed extracts in a number of tumor models including melanoma, nasopharyngeal, gastric carcinoma, breast cancer etc. [13]–[18]. However, these studies are confined to polysaccharides and/or crude extracts, and the benefit of seaweed polyphenols or their active components against pancreatic carcinogenesis and/or its progression (if not for any tumor) has never been investigated. Furthermore, an in-depth insight on the effect of seaweed polyphenols in this setting, their potential in the regulation of tumor cell signaling, mode of action (potential yet definite molecular targets, if any), remains to be reconnoitered. Owing to the fact that the polyphenols target key cell signaling pathways, we investigated the effect of polyphenols from five different brown algae in PC settings. This study provides first-hand evidence on the antioxidant, anti-proliferative and cell killing potentials of polarity based extractions of these seaweeds. Further, this study also identifies the potential of the extracted polyphenols on major tumor progression molecular targets including NFκB, EGFR, kRAS, STAT3, VEGF, AKT, TERT, FGFA, BCl2 and PDGFA.

## Materials and Methods

### Cell Culture

Human Panc-1 (ATCC–CRL1469), Panc-3.27 (ATCC-CRL2549), BxPC-3 (ATCC-CRL1687), and MIA PaCa-2 (ATCC-1420) cells were obtained from Dr Daniel J. Brackett (Department of surgery, University of Oklahoma Health Sciences Center, Oklahoma City, OK). Culture and maintenance of Panc-1, BxPC-3, and MIA PaCa-2 cells were performed as described earlier [19]. Panc-3.27 cells were maintained as monolayer cultures by weekly serial passage in 100 mm tissue culture plates in high glucose RPMI-1640 medium with 2 mM L-glutamine, 10 mM HEPES, 1 mM sodium pyruvate, 1500 mg/L sodium bicarbonate, 10Units/ml human recombinant insulin and 15% fetal bovine serum. MCF10A (ATCC-CRL10317), Human aortic smooth muscle cells (HASMC, ATCC-PCS-100-012) and Human iliac artery endothelial cells (HIAE, ATCC) cells were obtained from Dr. Mohan Natarajan (University of Texas Health Sciences Center at San Antonio, TX). Cells were maintained as monolayer cultures in Medium 199 (Life Technologies, Grand Island, NY) supplemented with 2 mM L-glutamine, 1 mM sodium pyruvate, 1500 mg/L sodium bicarbonate, MEM vitamins, Non-Essential Amino Acids, pen/strep and 10% HIFBS. For MCF10A the medium was additionally supplemented with Mammary Epithelial Growth Supplement (Life Technologies) and cholera toxin (Sigma). For passage and for all experiments, the cells were detached using trypsin (0.25%)/EDTA (1%), re-suspended in complete medium, counted electronically using Countess automated cell counter (Carlsbad, CA), and incubated in a 95% air/5% CO2 humidified incubator.

### Polyphenol extraction and cell treatments

Cells plated in 100 mm tissue culture plates containing 6 ml of complete growth medium were allowed to grow up to 70% to 80% confluence. Five species of brown algae, *Dictyota dichotoma* (DD), *Hormophysa triquerta* (HT), *Spatoglossum asperum* (SA), *Stoechospermum marginatum* (SM) and *Padina tetrastromatica* (PT) were collected from the Mandapam coast, Gulf of Mannar, South east coast of India. Seaweeds are collected as a part of the Department of Science and Technology, Government of India sponsored DBT project and, since this collection does not involve any endangered or protected species, no specific permissions were required (Forest Department, Government of India exempt). Freshly collected algae were washed, oven dried and their powder was used for sequential extraction of polyphenols in gradient (increasing) polarity solvents including hexane (H), dichloromethane (DCM), ethyl acetate (EA) and methanol (M). In brief, 50 g of powdered algae soaked in three volumes of specific solvent was shaked for 48 h at room temperature. The slurry was filtered through Whatman filter paper followed by incubation of the residue in the subsequent solvent. Resulting four filtrates were completely dried in pre-weighed micro centrifuge tubes using SpeedVac (Thermo scientific, Asheville, NC). The dried filtrates were weighed and dissolved in dimethyl sulfoxide (DMSO) to a final stock concentration of 100 mg/ml. For cell treatment, polyphenol ‘‘stock’’ solutions were further diluted in plain cell culture (DMEM) medium to a ‘‘working’’ concentration of 10 mg/ml. Cells were treated with varying concentrations (10, 50, 100 µg/ml) of polyphenols and allowed to incubate at 37°C for 3 h and 24 h unless or otherwise mentioned in the text. The final concentration of DMSO in the cell culture medium was <0.0001%.

### Total antioxidant capacity analysis

To determine the antioxidant potential of 20 polyphenol fraction comprising of 4 characteristic fractions from each seaweed from a total of 5 seaweeds, total antioxidant capacity analysis (TCA) was performed as described by Prieto and colleagues [20]. In brief, two parts of each extraction at various concentrations (100, 250, 500 µg/ml and 1 mg/ml) was mixed with one part of TCA reagent (0.6 M sulfuric acid, 28 mM sodium phosphate and 4 mM ammonium molybdate) and incubated at 95°C for 90 min. Absorbance was measured at 635 nm using Synergy II micro plate reader (BioTek Instruments, Inc. Winooski, VT). Gallic acid (GA) was used as the positive control and negative controls with plain solvents were also included. Fractions that exhibit high levels of total anti-oxidant capacity will be utilized for further (anti-PC) analysis.

### Cell Viability

Trypan blue exclusion assay was used to identify the efficacy of seaweed polyphenols in the regulation of PC cell viability. Mia-PaCa-2, Panc-1, Panc-3.27 and BxPC-3 cells treated with varying concentrations (10, 20, 50 or 100 µg/ml) of dichloromethane fractions (DD-DCM, SA-DCM, SM-DCM, PT-DCM, HT-DCM) or ethyl acetate fractions (DD-EA, SA-EA, SM-EA, PT-EA, HT-EA) were examined after 24 h for alterations in cell viability. Quantitative assessment was made using the Countess automated cell counter as described earlier [19] and compared with untreated controls using two-way ANOVA with Bonferonii post-hoc test (GraphPad PRISM v4.03, GraphPad Prism Software Inc., La Jolla, CA). A P value of <0.05 is considered statistically significant.

### Cell survival

Cell survival was analyzed using CyQUANT Cell Proliferation Assay Kit (Molecular Probes, Inc. Eugene, OR, USA) in Panc-1 and MIA PaCa-2 cells treated with dichloromethane (DD-DCM, SA-DCM, SM-DCM, PT-DCM, HT-DCM) or ethyl acetate (DD-EA, SA-EA, SM-EA, PT-EA, HT-EA) fractions following manufacturer's protocol. In brief, cells (5,000 cells/well) seeded in 96-well plate with 200 µl/well growth medium was incubated at 37°C for a period 24 h. For dose escalation studies cells were exposed to increasing concentrations (10, 20, 50 and 100 µg/ml) of seaweed polyphenols and analyzed after 3 h. For time-dependent recovery studies, the cells were treated with 100 µg/ml of polyphenol fractions and examined after 3, 24, 48 or 72 h. After treatment, the growth medium was carefully and completely removed and the plates were kept under freezing (−70°C) for additional 24 h. Frozen cells were then lysed with CyQUANT® GR dye/cell-lysis buffer (200 µL/well) at room temperature, protected from light for 5 min and the fluorescence (480 nm excitation and 520 nm emission) using Synergy II micro plate reader (BioTek). All samples were assayed in triplicate. Negative controls with no cells were included. Group-wise comparisons were made using two-way ANOVA with Bonferonii post-hoc test (GraphPad PRISM) and a P value of <0.05 is considered statistically significant. Further, to determine whether this polyphenol(s) driven anti-proliferation is tumor-cell selective and, to delineate their normal cell cytotoxicity, if any, we examined their effects on primary cells. For this, MCF10A, HIAE and HASMC cells treated with 100 µg/ml of DD-DCM, SA-DCM, SM-DCM, PT-DCM, HT-DCM, DD-EA, SA-EA, SM-EA, PT-EA or HT-EA were examined after 24 h for alterations in cell proliferation.

### Nuclear morphology by dual staining

Panc-1, Panc-3.27, BxPC-3 and Mia-PaCa-2 cells (5×10^5^ cells) grown in 4-well plate (Nunc) treated with 100 µg/ml of dichloromethane (DD-DCM, SA-DCM, SM-DCM, PT-DCM, HT-DCM) or ethyl acetate (DD-EA, SA-EA, SM-EA, PT-EA, HT-EA) fractions were analyzed after 24 h for nuclear morphology as described earlier [21].

### DNA Fragmentation on Agarose Gels

DNA laddering assay was used to identify the efficacy of seaweed polyphenols in inducing PC cell DNA fragmentation. In brief, DNA from Panc-1, Panc-3.27, BxPC-3 and Mia-PaCa-2 cells treated with DD-DCM, SA-DCM, SM-DCM, PT-DCM, HT-DCM, DD-EA, SA-EA, SM-EA, PT-EA and HT-EA for 24 h was extracted with DNA stat-60 (Tel-Test Inc., Friendswood, TX, USA) following manufacturer's instruction. DNA samples (5 µg) were electrophoresed in 2% agarose gel containing ethidium bromide, visualized using ChemiDoc™ XRS imager (Biorad, Hercules, CA) and quantified using Quantity-One image analysis software (Biorad). Group-wise comparisons were made using analysis of variance with Tukey's post-hoc correction. A P value of <0.05 is considered statistically significant.

### Plasmid Preparation, DNA Transfection, and Luciferase Reporter Assay

Alterations in NFκB promoter activation in dichloromethane (DD-DCM, SA-DCM, SM-DCM, PT-DCM, HT-DCM) or ethyl acetate (DD-EA, SA-EA, SM-EA, PT-EA, HT-EA) fractions treated Panc-1, BxPC-3 and MiaPaCa-2 cells were investigated using luciferase reporter assay. The pNFκB-Luc plasmid construct was amplified and purified as described in our earlier studies [22], [23]. Cell lysates from cell treated for 24 h were assayed for luciferase activity as per the manufacturer's protocol (Biovision Research Products, Mountain View, CA) and the group wise comparisons were made using ANOVA with Tukey's post-hoc correction. A P value of <0.05 is considered statistically significant.

### QPCR

Transcriptional alterations of key tumor progression molecules including, *Bcl2, EGFR, PDGFA, VEGF, AKT, TERT, kRAS and FGF* in dichloromethane (DD-DCM, SA-DCM, SM-DCM, PT-DCM, HT-DCM) or ethyl acetate (DD-EA, SA-EA, SM-EA, PT-EA, HT-EA) fractions treated (for 3 h) Panc-1, Panc-3.27, BxPC-3 and MiaPaCa-2 cells were analyzed by real-time QPCR as described earlier [23]. We used β*-actin* as a positive control, and a negative control without template RNA was also included. Each experiment was carried out in triplicate, and the ^ΔΔ^ *C*t values were calculated by normalizing the gene expression levels to β*-actin*, and the relative expression level was expressed as a fold change.

### Immunoblotting

Panc-1, BxPC-3 and MiaPaCa-2 cells exposed to dichloromethane (DD-DCM, SA-DCM, SM-DCM, PT-DCM, HT-DCM) or ethyl acetate (DD-EA, SA-EA, SM-EA, PT-EA, HT-EA) fractions were analyzed after 24 h for the alterations in the phosphorylation of EGFR and protein levels of kRAS, STAT3, EGFR and AURKβ. Total protein extraction and immunoblotting were performed as described in our earlier studies [23], [24]. For this study, the protein-transferred membranes were incubated either with rabbit monoclonal anti-pEGFR^(Tyr1068)^ (Cell Signaling Technology, Inc., Danvers, MA, USA), kRAS (Proteintech Group, Inc. Chicago, IL, USA), STAT3 (Santa Cruz Biotechnology, Inc, Santa Cruz, CA, USA) or mouse monoclonal EGFR (Santa Cruz), AURKβ antibody (Proteintech). The blots were stripped and reblotted with mouse monoclonal anti-α-tubulin antibody (Santa Cruz) to determine equal loading of samples. Densitometry analysis was performed using Quantity-One (Biorad) and the relative band intensity was plotted in a histogram using PRISM 5 (GraphPad Software, Inc., La Jolla, CA, USA). Group wise comparisons were made using analysis of variance with Tukey's post-hoc correction.

## Results

### Seaweed polyphenols harbor high levels of antioxidants

**Figure 1:**
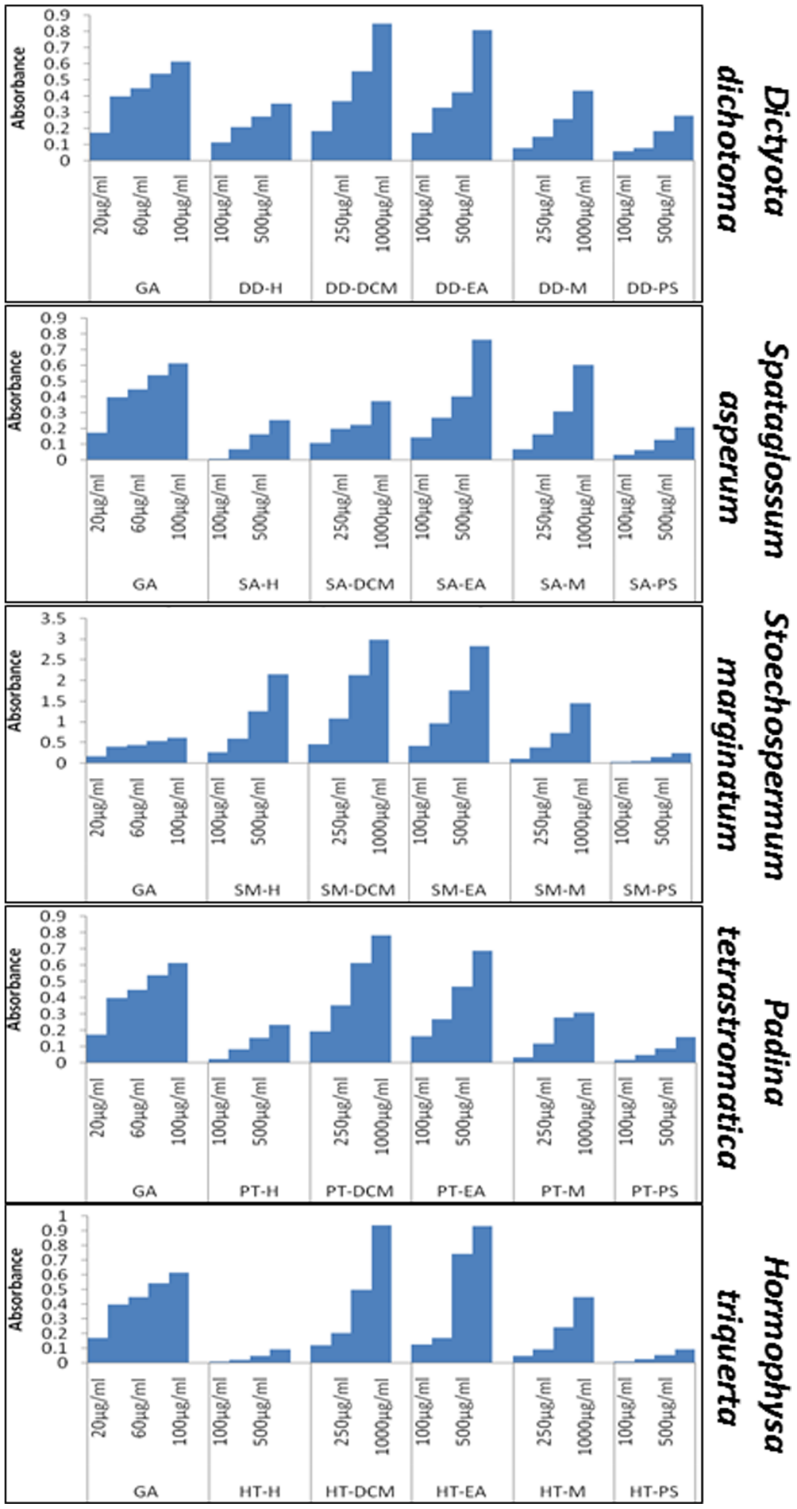
Histogram(s) showing total antioxidant levels in seaweed polyphenol fractions. The polyphenols were extracted from five species of brown algae, *Dictyota dichotoma* (DD), *Hormophysa triquerta* (HT), *Spatoglossum asperum* (SA), *Stoechospermum marginatum* (SM) and *Padina tetrastromatica* (PT) using gradient polarity solvents including hexane (H), dichloromethane (DCM), ethyl acetate (EA) and methanol (M). Total antioxidant capacity analysis was performed with Gallic acid (GA) as positive control, plain solvents as negative controls and polysaccharide fraction from corresponding seaweed as relative measure.

To determine the antioxidant potentials of seaweed polyphenols, we examined the total antioxidant capacity of extracted fractions including hexane-, dichloromethane-, ethyl acetate- and methane- fractions of all five seaweeds investigated. Gallic acid was used as the positive control and a polysaccharide fraction from corresponding seaweed was also examined to provide a relative magnification of antioxidants in polyphenols. TCA revealed significant levels of antioxidants in all seaweed polyphenol fractions (Fig. 1). Dose-dependent increase in the antioxidant levels of all the fractions investigated precisely validates antioxidant availability and concentration. Relatively, we observed very high levels of antioxidants in dichloromethane and ethyl acetate fractions of all seaweeds investigated (Fig. 1). Inimitably, polyphenols extracted from *Stoechospermum marginatum* showed to harbor maximum antioxidants, ∼3.5 fold higher in DCM and EA fractions (Fig. 1). Owing to the fact that, the DCM and EA fractions contains high antioxidants across the brown algae examined, we selectively utilized these two fractions from each sea weed to delineate the anti-PC potential.

### Antioxidant-rich polyphenol fractions retards PC cell viability

**Figure 2:**
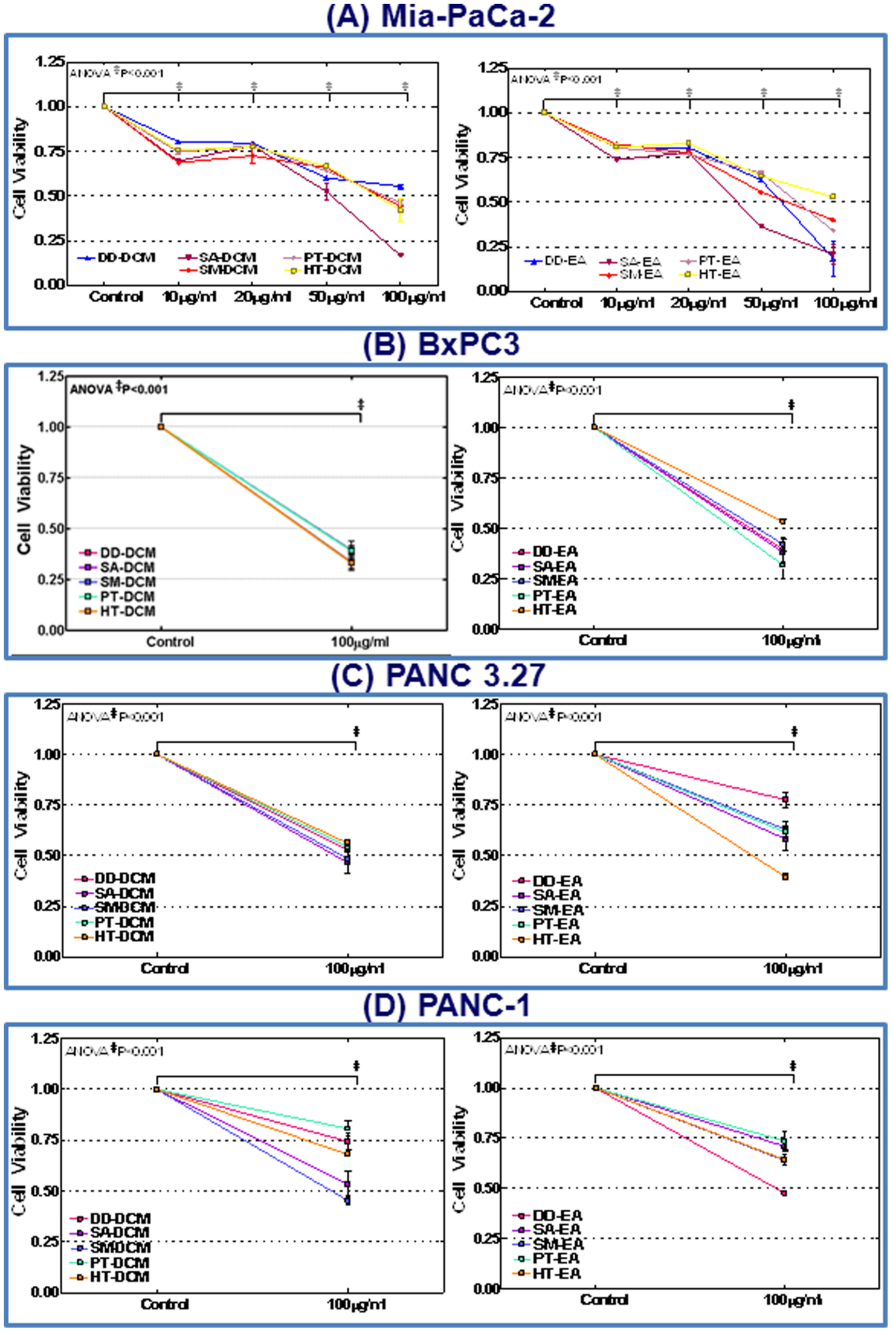
Line plots of trypan blue exclusion assay showing dose-dependent inhibition of cell viability with 10, 20, 50 and 100 µg/ml of dichloromethane (DD-DCM, SA-DCM, SM-DCM, PT-DCM, HT-DCM) or ethyl acetate (DD-EA, SA-EA, SM-EA, PT-EA, HT-EA) fractions after 24 h in (A) MiaPaCa-2 cells. Dose associated regulation of cell viability for each polyphenol fractions were compared to untreated controls using two-way ANOVA with Bonferonii post-hoc test and a P value of <0.05 is considered statistically significant. Line plots showing significant inhibition of cell viability in (B) BxPC-3, (C) Panc-3.27 and (D) Panc-1 cells treated with 100 µg/ml DCM or EA fractions.

Mia-PaCa-2, Panc 3.27, Panc 1 and BxPC3 cells treated with dichloromethane (DD-DCM, SA-DCM, SM-DCM, PT-DCM, HT-DCM) or ethyl acetate (DD-EA, SA-EA, SM-EA, PT-EA, HT-EA) fractions (10, 20, 50 or 100 µg/ml) were examined for alterations in cell viability using Trypan blue exclusion assay. All DCM and EA polyphenol fractions showed a significant (P<0.001) inhibition of cell viability as low as 10 µg/ml concentration (Fig. 2A). With increasing concentrations of polyphenol fractions, we observed a dose dependent inhibition of cell viability in MiaPaCa-2 cells exposed to DCM or EA fractions (Fig. 2A). Consistently, 100 µg/ml of all DCM and EA fraction(s) significantly introverted cell viability (>60%) in BxPC3 cells (Fig. 2B). However, HT-EA showed relatively less inhibitory potential in this cell line. Like-wise, we observed a consistent inhibitory efficiency (∼50%) of all DCM fractions in Panc 3.27 cells (Fig. 2C). On the other hand, EA fractions showed a slope in efficacy from low-activity with DD-EA to a maximal-activity with HT-EA in Panc 3.27 cells (Fig. 2C). In Panc 1 cells, both DCM and EA fractions markedly inhibited cell viability (Fig. 2D). Relatively, we observed a maximum inhibition of cell viability with SM-DCM and DD-EA (>50%) fractions. These results demonstrate that seaweed polyphenols potentially inhibit PC cell viability and further identify the ‘*cell-type-*independent’ minimal efficient (50%) inhibitory dose in this setting.

### Seaweed polyphenols inhibits PC cell proliferation

**Figure 3:**
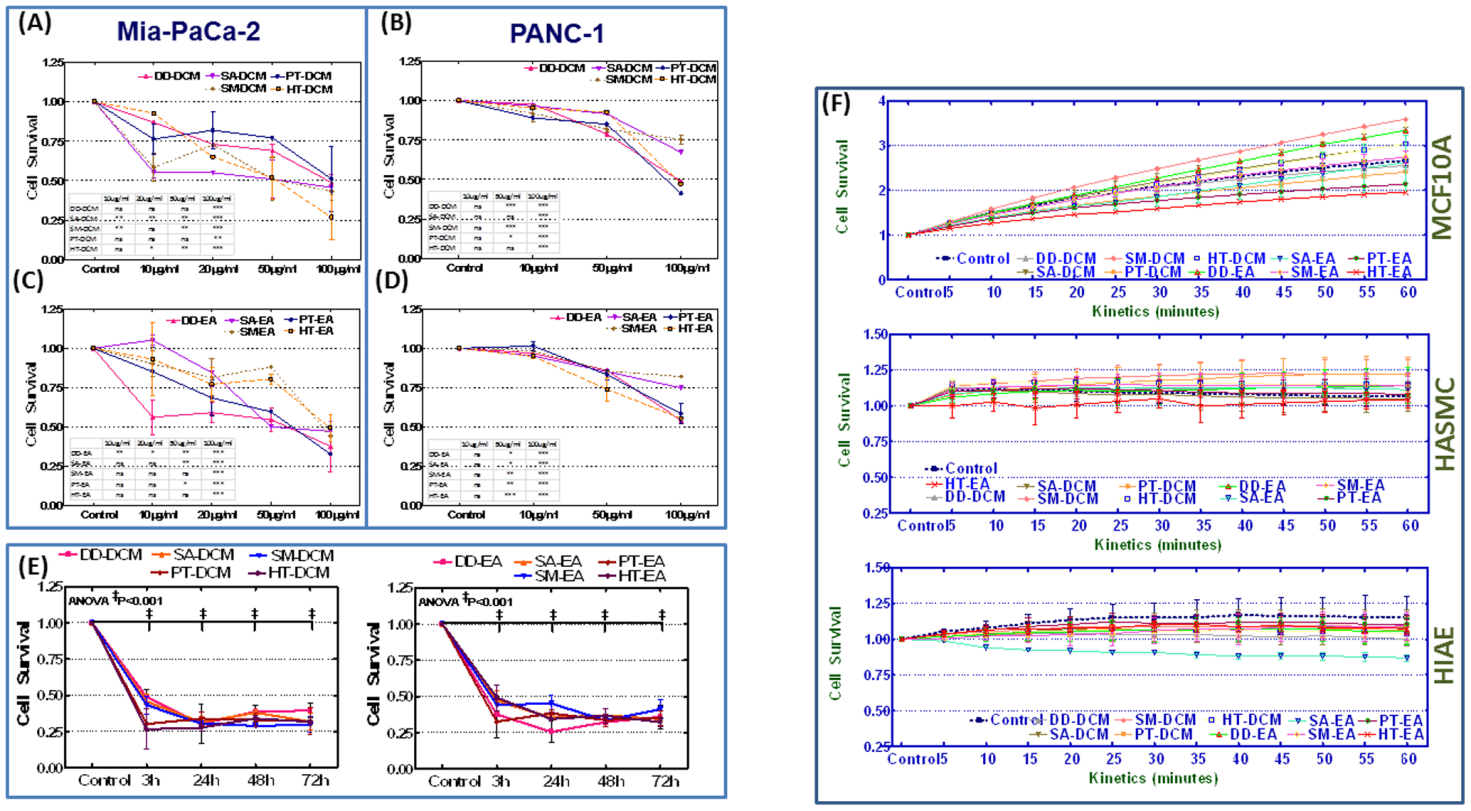
Line plots of CyQUANT-NF cell proliferation assay showing dose dependent inhibition of cell survival with 10, 20, 50 and 100 µg/ml of (A & B) dichloromethane (DD-DCM, SA-DCM, SM-DCM, PT-DCM, HT-DCM) fractions or (C & D) ethyl acetate (DD-EA, SA-EA, SM-EA, PT-EA, HT-EA) fractions in MiaPaCa-2 and Panc-1 cells. The cells exposed to the seaweed polyphenols were assessed for cell proliferation 3 h post treatment. Dose associated inhibition of cell proliferation for each polyphenol fractions were compared to untreated controls using two-way ANOVA with Bonferonii post-hoc test and a P value of <0.05 is considered statistically significant. (E) Line plots of DD-DCM, SA-DCM, SM-DCM, PT-DCM, HT-DCM, DD-EA, SA-EA, SM-EA, PT-EA and HT-EA exposed MiaPaCa-2 cells showing significant and sustained (3, 24, 48 and 72 h) inhibition of cell proliferation. (F) Line plots of DD-DCM, SA-DCM, SM-DCM, PT-DCM, HT-DCM, DD-EA, SA-EA, SM-EA, PT-EA and HT-EA exposed normal human MCF10A, HASMC and HIAE cells showing consistent cell survival 24 h after treatment. Compared to untreated controls, all DCM and EA fractions revealed no significant inhibition of cell proliferation all three cell lines investigated. Time-line in the line-plots shows the fluorescence (480 nm excitation and 520 nm emission) measurements continuously up to 60 minutes.

Further to substantiate the cell-viability inhibitory potential of anti-oxidant rich polyphenols indeed translates to the consequent regulation of cell survival, we examined the anti-proliferative potential of DCM and EA fractions. To achieve this, we measured the total DNA content (that closely proportional to cell number) and the extent of proliferation was determined by comparing cell counts of drug treated MiaPaCa-2 and Panc-1 cells with untreated controls. Compared to the untreated controls, CyQuant-NF analysis revealed a significant dose- (10, 20, 50 and 100 µg/ml) dependent inhibition of cell proliferation both in MiaPaCa-2 and Panc-1 cells with each dichloromethane (DD-DCM, SA-DCM, SM-DCM, PT-DCM, HT-DCM) or ethyl acetate (DD-EA, SA-EA, SM-EA, PT-EA, HT-EA) fractions (Fig.3). However, these anti-oxidant rich fractions exhibited a ‘cell-type dependent’ and/or ‘solvent-dependent’ magnitude of cell proliferation inhibition. Evidently, all five DCM fractions (100 µg/ml) profoundly (∼50%) inhibited MiaPaCa-2 cell proliferation with maximum (∼75%) inhibition observed after HT-DCM treatment (Fig. 3A). Like-wise, in Panc-1 cells, DD-DCM, PT-DCM and HT-DCM treatment robustly (∼50%) inhibited cell proliferation with maximum inhibition in PT-DCM treated cells (Fig. 3B). More importantly, all EA fractions also exhibited DCM comparable (∼50%) cell proliferation inhibition potential in MiaPaCa-2 cells (Fig. 3C). Evidently, PT-EA treatment displayed maximum inhibition in these cells. Though, DD-EA, PT-EA and HT-EA showed inhibitory potential in Panc-1 cells, relatively (as opposed to DCM), EA fractions produced marginal effect (Fig. 3D). Secondly, we examined whether the seaweed polyphenol inhibited cell-survival is transient (with time dependent recovery) or a permanent cause effect. MiaPaCa-2 cells treated with 100 µg/ml of DCM and EA fractions were subjected to cyquant analysis after 3, 24, 48 and 72 h. Compared to untreated controls, seaweed polyphenols exerts a significant (P<0.001) anti-proliferation as low as 3 h (Fig. 3E). More importantly, this DCM and EA fractions induced inhibition of cell proliferation remained consistent after 24, 48 and 72 h (Fig. 3E). These results demonstrate the anti-proliferative potential of seaweed polyphenols in PC cells and further short-list the potential fractions in this setting.

Further to determine the tumor selective potential of seaweed polyphenols and to delineate normal cell cytotoxicity, if any, we examined the effects of these fractions on healthy cell survival. For this MCF10A, HIAE and HASMC cells treated with 100 µg/ml of DD-DCM, SA-DCM, SM-DCM, PT-DCM, HT-DCM, DD-EA, SA-EA, SM-EA, PT-EA or HT-EA were examined after 24 h for alterations in cell proliferation. Fluorescence (480 nm excitation and 520 nm emission) was measured continuously up to 60 minutes. Compared to untreated controls, CyQuant NF analysis did not reveal any significant inhibition of cell survival in MCF10A cells. Like-wise, we observed a consistent cell survival both in HASMC and HIAE cells treated with DD-DCM, SA-DCM, SM-DCM, PT-DCM, HT-DCM, DD-EA, SA-EA, SM-EA, PT-EA or HT-EA implying no definite cytotoxicity of these fractions in normal cells.

### Seaweed polyphenols exerts apoptotic cell death in PC cells

**Figure 4:**
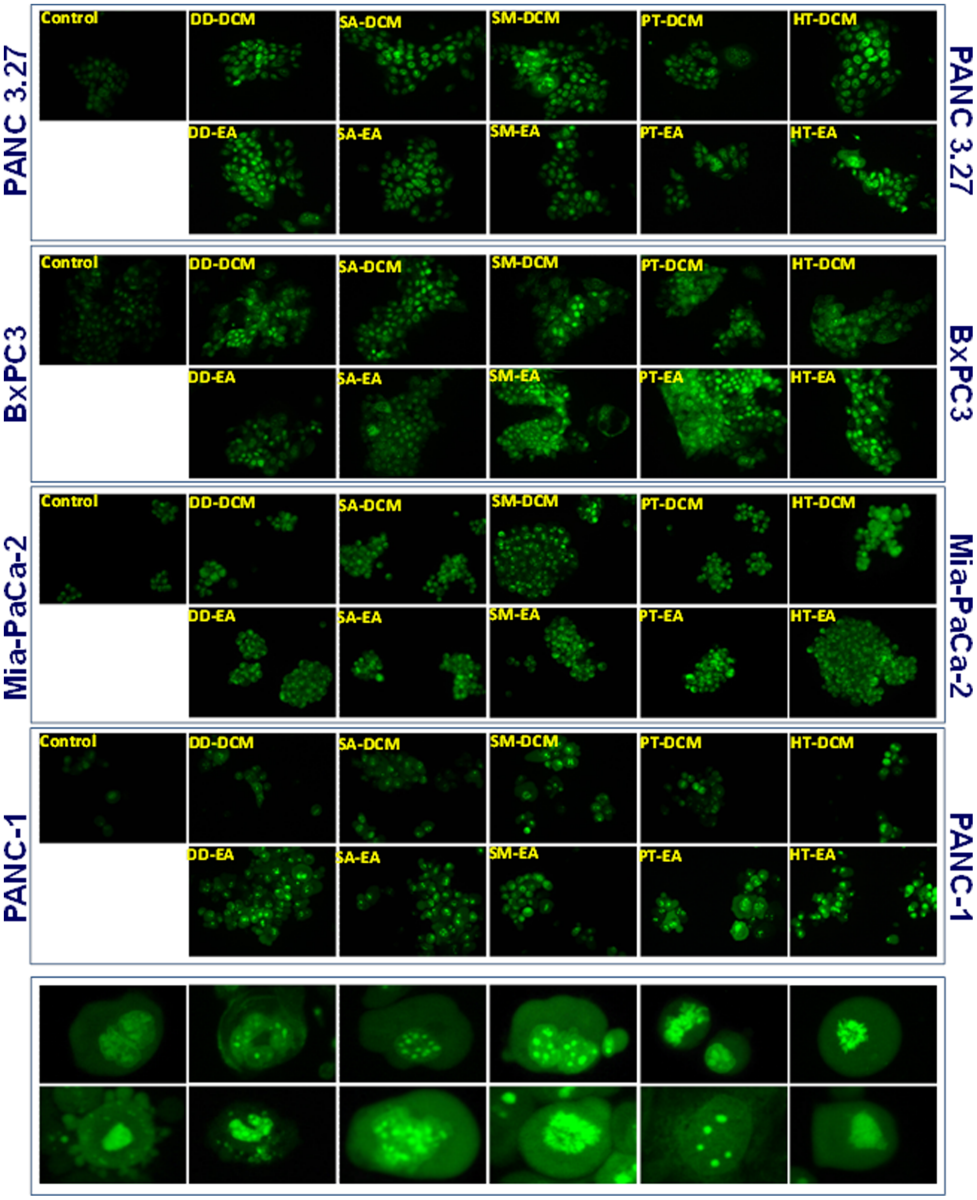
Nuclear morphology with dual staining showing apoptotic characteristics in Panc-3.27, BxPC-3, MiaPaCa-2 and Panc-1 cells treated with dichloromethane (DD-DCM, SA-DCM, SM-DCM, PT-DCM, HT-DCM) and ethyl acetate (DD-EA, SA-EA, SM-EA, PT-EA, HT-EA) seaweed polyphenol fractions for 24 h. Insert: High magnification photomicrographs showing chromatin with blebbing, nuclear condensation, and fragmentation indicating typical apoptotic characteristics in cells treated with seaweed polyphenols.

**Figure 5:**
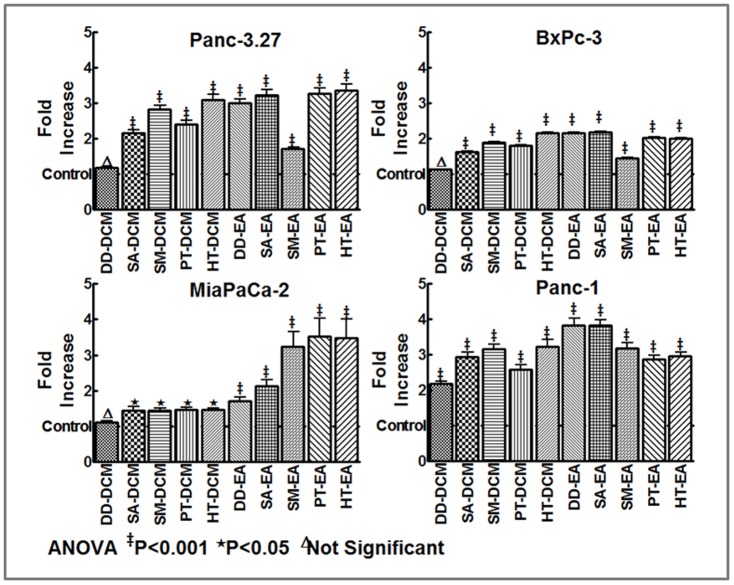
Histograms showing DNA fragmentation in Panc-3.27, BxPC-3, MiaPaCa-2 and Panc-1 cells treated with dichloromethane (DD-DCM, SA-DCM, SM-DCM, PT-DCM, HT-DCM) and ethyl acetate (DD-EA, SA-EA, SM-EA, PT-EA, HT-EA) seaweed polyphenol fractions and harvested after 24 h. DNA samples were electrophoresed in 2% agarose gel containing ethidium bromide and quantified using Quantity-One.

To delineate whether the anti-PC potential of seaweed polyphenols involve apoptotic cell death and to identify the potential magnitude of different fractions characterized, we examined the associated alterations in DNA fragmentation. Since assessment of apoptosis cannot be relied on one single end point, we utilized both qualitative acridine orange/ethidium bromide staining and semi-quantitative agarose gel DNA fragmentation in Panc-1, Panc 3.27, BxPC3 and MiaPaCa-2 cells. Compared to untreated controls, fluorescence microscopy revealed that all anti-oxidant rich polyphenol fractions including DD-DCM, SA-DCM, SM-DCM, PT-DCM, HT-DCM, DD-EA, SA-EA, SM-EA, PT-EA and HT-EA exhibits apoptotic characteristics (Fig. 4). Evidently, we observed differential DNA fragmentation magnitudes across four PC cell lines treated with these polyphenols. Consistently, compared to the untreated controls, agaorse gel-DNA fragmentation analysis showed statistically significant (P<0.05) induction of DNA fragmentation in Panc 1, Panc 3.27, BxPC-3 and MiaPaCa-2 cells treated with seaweed polyphenols, except for DD-DCM (Fig. 5). Comparatively, we observed a maximal (>2 fold) DNA damaging effect of all seaweed polyphenols in Panc-1 and Panc-3.27 cells. Extent of DNA fragmentation inflicted by these polyphenols demonstrates that the seaweeds employ apoptotic cell-killing in PC.

### Seaweed polyphenols inhibits functional activation of NFκB

**Figure 6:**
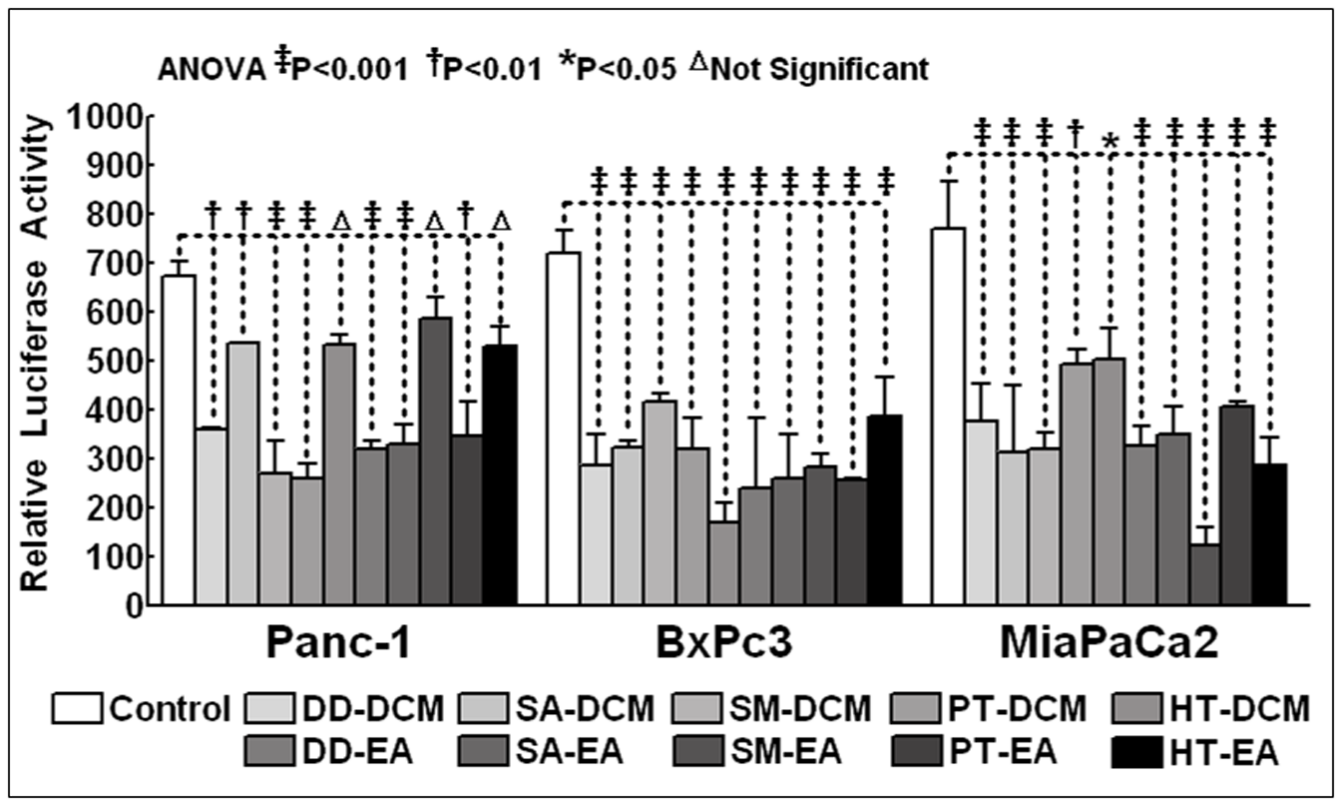
Luciferase assay showing seaweed polyphenols inhibits functionally activated NFκB. Panc-1, BxPC-3 and MiaPaCa-2 cells were transfected with pNFκB-Luc and then treated with or without 100 µg/ml of dichloromethane (DD-DCM, SA-DCM, SM-DCM, PT-DCM, HT-DCM) and ethyl acetate (DD-EA, SA-EA, SM-EA, PT-EA, HT-EA) seaweed polyphenol fractions. Cells were then harvested at 24 h post-treatment. Data shown represent mean and standard deviation (SD) of three independent experiments. Significant decrease in luciferase activity in seaweed polyphenols treated PC cells.

Further to investigate that the seaweed polyphenols induced apoptotic cell death involves the inhibition of pro-survival NFκB, we examined the regulation of NFκB promoter activation with these DCM and EA fractions in PC cells. For this, human Panc-1, BxPC-3 and MiaPaCa-2 cells were transiently transfected with a pNFκB-Luc plasmid construct that expresses the luciferase reporter gene in an NFκB-dependent manner. Binding of NFκB to the promoter activates transcription, allowing the Luc reporter gene to be expressed. Transfected cells were exposed to DCM and EA fractions (individually), and the luciferase reporter assay was performed after 24 h. Cells treated with seaweed polyphenol DCM and EA fractions resulted in profound inhibition in luciferase activity, indicating that these fractions could specifically alleviate transcriptional activation of NFκB (Fig. 6). Though we found marginal differences between fractions and across cell-lines, in general the data clearly portraits a remarkable reduction of luciferase activity much lesser than the basal levels demonstrating their potential in attenuating NFκB transcription.

### Seaweed polyphenols regulate tumor progression molecules in PC cells

**Figure 7:**
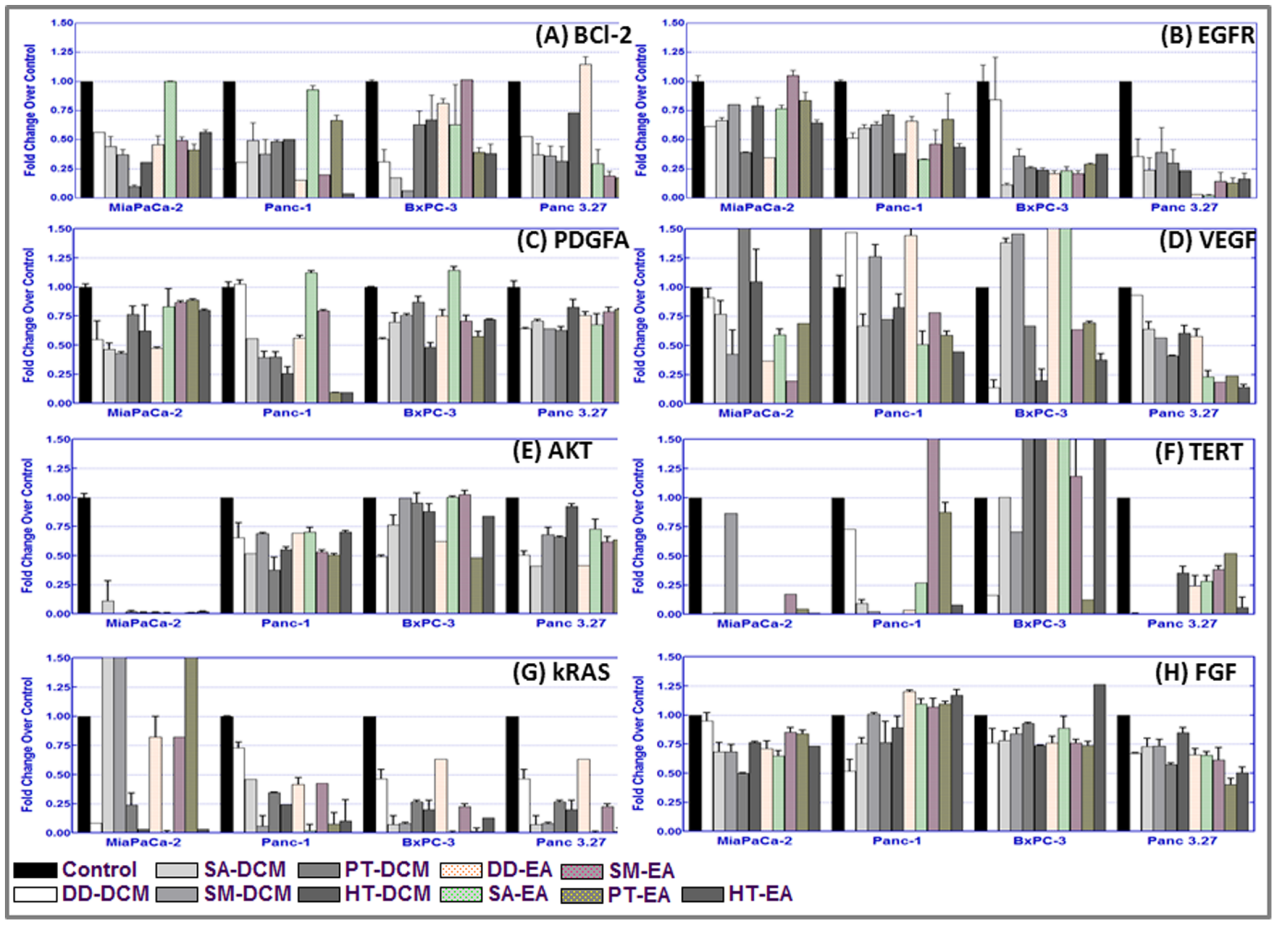
QPCR analysis showing transcriptional levels of (A) *BCl2*, (B) *EGFR*, (C) *PDGFA*, (D) *VEGF*, (E) *AKT*, (F) *hTERT*, (G) *kRas* and (H) *FGF* mRNA expression in MiaPaCa-2, Panc-1, BxPC-3 and Panc-3.27 cells treated with 100 µg/ml of dichloromethane (DD-DCM, SA-DCM, SM-DCM, PT-DCM, HT-DCM) and ethyl acetate (DD-EA, SA-EA, SM-EA, PT-EA, HT-EA) seaweed polyphenol fractions and harvested after 3 h post-treatment.

**Figure 8:**
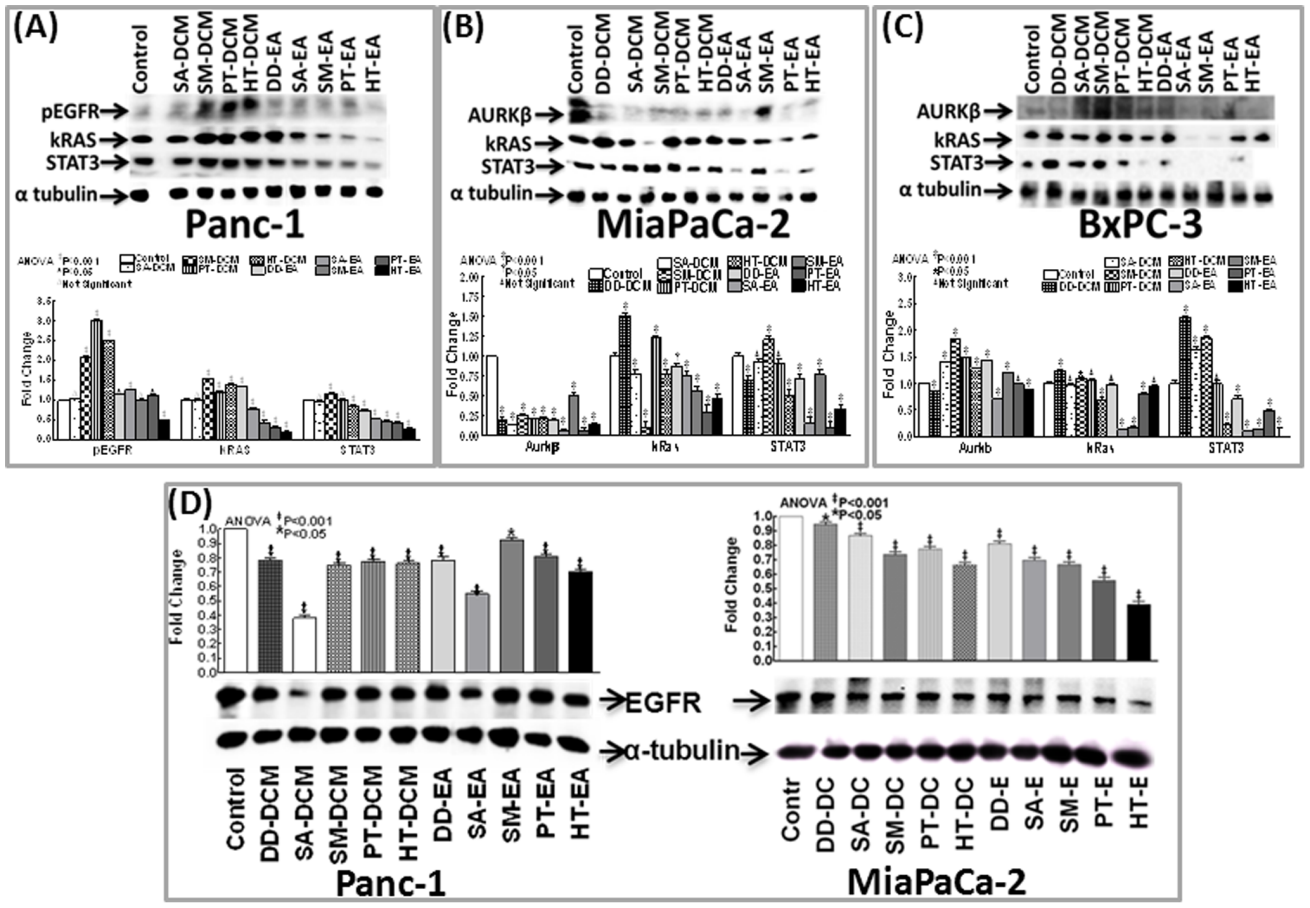
Representative immunoblots showing modulation in the (A) phosphorylation of EGFR, protein levels of kRas, STAT3 in Panc-1, Aurkβ, kRAS, STAT3 in (B) MiaPaCa-2 and (C) BxPC-3 cells treated with 100 µg/ml of dichloromethane (DD-DCM, SA-DCM, SM-DCM, PT-DCM, HT-DCM) and ethyl acetate (DD-EA, SA-EA, SM-EA, PT-EA, HT-EA) seaweed polyphenol fractions and analyzed after 24 h. (D) Representative immunoblots showing modulation in the protein levels of EGFR in Panc-1 and MiaPaCa-2 cells treated with 100 µg/ml of DCM and EA fractions and analyzed after 24 h. α-tubulin was used to validate equal loading of samples. Histograms showing the Quantity-One densitometry of relative band intensities. Significance of expression was assessed by comparing each drug treatment to the untreated control of each cell line.

Also, to dissect out the benefit of anti-oxidant rich seaweed polyphenols in targeting tumor progression markers, first, we investigated the transcriptional regulation of *Bcl2, EGFR, PDGFA, VEGF, AKT, TERT, kRas* and *FGF* in PC cells. MiaPaCa-2, Panc-1, Panc-3.27 and BxPC-3 cells treated with DCM (DD-DCM, SA-DCM, SM-DCM, PT-DCM, HT-DCM) or EA (DD-EA, SA-EA, SM-EA, PT-EA, HT-EA) fractions for 3 h were analyzed for mRNA alterations using QPCR (Figure S1). β-actin normalized expression compared as fold change over untreated controls revealed a differential, yet, unique transcriptional modulation of *Bcl2, EGFR, PDGFA, VEGF, AKT, TERT, kRas* and *FGF* in between fractions and across four PC cell lines investigated (Fig. 7). In general, seaweed fractions alleviate (as opposed to untreated controls) the transcription of these molecules in the PC. Further, immunoblotting analysis revealed that the seaweed polyphenols differentially regulate the phosphorylation of EGFR and protein levels of EGFR, kRAS, STAT3 and AurKβ in Panc-1, MiaPaCa-2 and BxPC-3 cells (Fig. 8). It is noteworthy to mention that while pEGFR levels remain almost unaltered, the expression of total EGFR correlated well with the transcription data. Fractions associated distinction in the regulation of these tumor progression markers/oncogenes demonstrate that each fractions may target multiple (alternative), cell-type-dependent signaling in PC.

## Discussion

The results of this screening experiment demonstrated that the sequential polarity (hexane, dichloromethane, ethyl acetate and methanol) based extraction of polyphenols of brown algae, *Dictyota dichotoma* (DD), *Hormophysa triquerta* (HT), *Spatoglossum asperum* (SA), *Stoechospermum marginatum* (SM) and *Padina tetrastromatica* (PT) collected from the Mandapam coast, Gulf of Mannar, South east coast of India possessed diverse anti-oxidant properties. The type of solvent had great impact on total anti-oxidant capacity of seaweeds. Dichloromethane and ethyl acetate fractions from all five seaweeds harbor relatively high levels of TCA than hexane and methanol extracted polyphenols. A plethora of evidence has shown a tight association between high anti-oxidant capacity and anti-cancer properties of a number of phytochemicals [25], [26] including seaweed polyphenols [6], [27]. In this perspective, we exploited high anti-oxidant harboring DCM and EA extracts from these five brown seaweeds to screen the anti-tumorigenic, in particular, anti-PC potential. Though a number of studies elucidated the anti-cancer potential of seaweeds across the globe, most of these studies constrained to specific seaweed, non-translatable fractionation, superficial screening and produced equivocal outcomes. More to that knowledge on the anti-tumorigenic capacity of polyphenols is few and fragmentary [28], [29]. A careful and sequential dissection from extraction -to- potential screening –to- characterization –to- selective potential screening –to- toxicity profiling – to- translatable animal model (distribution, bioavailability, potential, synergism, delivery modality) of the seaweeds, for that matter any phytochemicals, is an absolute ultimatum for translating a phytochemical to a clinical drug. Accordingly, this study focusses on the initial two-step, characteristic extraction –to- potential anti-tumor screening of the polarity based polyphenols of brown seaweeds. New to science, this study comprehensively recognizes the anti-PC potential and initial insight on the seaweed polyphenol specific targeting of potential molecular orchestration in PC cells.

As the genetic profile of PC is emerging with the present-day in-depth research, the role of specific genetic alterations that initiate tumorigenesis and subsequent cardinal clinical features of locally aggressive growth, metastasis, and chemotherapy resistance is gradually unveiling. To that end, a number of recent studies including studies from our group have shown that the inhibition of constitutive NFκB activation, one of the frequent molecular alterations in PC [30], [31], inhibits tumorigenesis [31]–[33], progression [34]–[36] and metastasis [19], [37], [38]. Inhibition of NFκB also sensitizes PC cell lines to chemotherapy and/or radiotherapy [39]–[43]. Owing to the crucial role of NFκB in multiple stages of PC, it has been considered a potential target for developing novel therapeutic strategies for the disease [31], [44], [45]. Armed with this background, we investigated whether the anti-oxidant rich seaweed polyphenols have any influence in the regulation of NFκB in PC cells. Since examining the alterations in constitutive levels, phosphorylation, nuclear translocation and even DNA-binding activity may not directly reflect the transcriptional potential, we utilized promoter-reporter approach to detect the seaweed associated influence on NFκB. Interestingly, our results recognized that all seaweed polyphenols alleviated NFκB in every PC cell lines investigated, signifying that indeed these seaweed polyphenols could be developed as potential deliverables to mitigate pancreatic carcinogenesis, tumor progression and metastasis.

**Figure 9:**
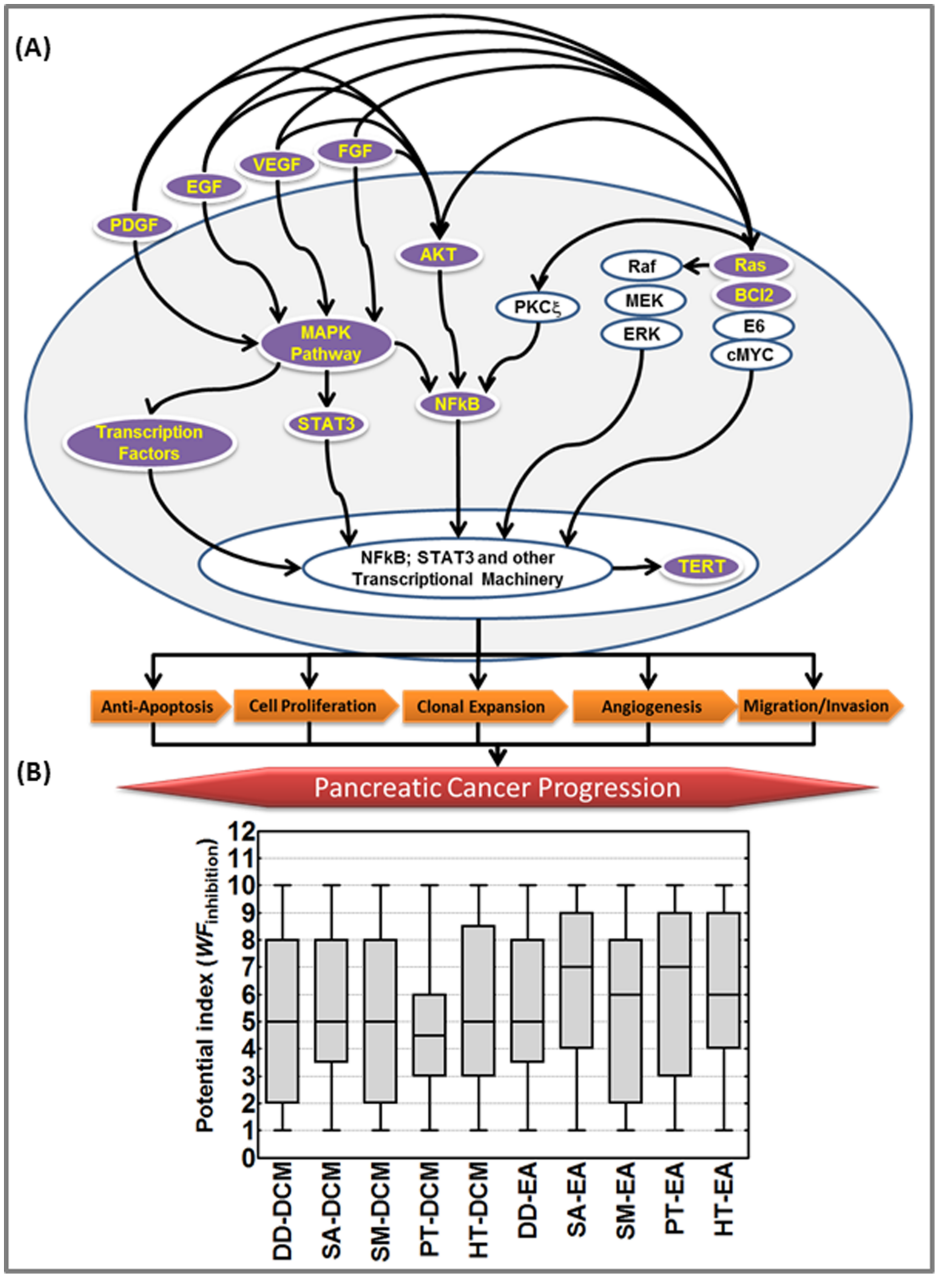
Schematic representation showing the (A) signaling pathway flow of specific upstream molecular mediators that triggers and promote PC progression. Seaweed polyphenols investigated in this study significantly alleviated the expression of these key molecular activators including EGFR, PDGFA, FGF, VEGFA, NFκB, STAT3 and TERT in PC cells. (B) A box and whisker plot showing the relative potential index for DD-DCM, SA-DCM, SM-DCM, PT-DCM, HT-DCM, DD-EA, SA-EA, SM-EA, PT-EA and HT-EA in human PC cells. The potential index for each seaweed polyphenol fractions were calculated by earmarking a weightage inhibition factor (*WF_inhibition_*) for every molecular endpoints measured (*Table S1*) from a range of 1 to 10, 10 being highest potential of inhibition. Calculated *WF_inhibition_* values were then plotted in a box-and-whisker plot with immediate visuals of the center, the spread, and the overall range of distribution. Box and whiskers plot identified three fractions SA-EA, PT-EA and HT-EA with *WF_inhibition_* of 9.0 upper quartile range.

Given the complex mutational profile of PC and the impossibility to target every single alteration, worldwide studies are attempting to understand the genes/pathways that are required for tumor maintenance [46]. Mutations in the kRas gene occur early during disease progression [47] and, the mouse model studies have shown a key role of mutant kRas in the initiation of this disease [48], [49]. In addition, it has been shown that human PC cell lines require kRas for growth and survival both *in vitro* and in immuno-compromised host mice [50], [51]. Like-wise, capacious evidence demonstrate that hTERT play an essential role in pancreatic tumorigenesis and progression and, even considered as a prognostic marker [52]–[54]. In this regard, a number of TERT silencing approaches proved beneficial in tumor regression. Evidently, knockdown of hTERT with siRNA suppresses the growth of PC cells via Bcl2 inhibition [55] and potentiate chemotherapy [56]. In addition, voluminous evidence implicate EGFR [57]–[60], *AKT* [*61*]–[*64*], AurKβ, STAT3 [65]–[68], PDGFA [69], [70], VEGF [71]–[73] and FGF [74]–[77] in pancreatic carcinogenesis, progression/metastasis and are comprehended as potential molecular targets to mitigate the disease. Inimitably, in this study, we observed a distinctive inhibitory profile of these key molecular targets including *Bcl2, EGFR, PDGFA, VEGF, AKT, TERT, kRas, FGF, STAT3 and Aurkβ*with each seaweed polyphenols tested in every cell-line investigated. To that end, it is noteworthy to mention that the molecular orchestration of these molecules are inter-related and play in unison (mostly bottle neck through transcriptional machinery) to promote anti-apoptosis, cell proliferation, clonal expansion, angiogenesis, invasion and migration (Fig. 9A). Ras activates many signaling cascades. Ras stimulation of the lipid kinase activity of PI3K stimulates the AKT/PKB kinase that inhibits apoptosis by inhibiting the actions of Bad, Caspase9 and AFX and, though phosphorylating the IκB repressor of NFκB. Ras also stimulates the mitogen-activated kinases ERK1/2 via the Raf1 cascade. The Erk kinases translocate to the nucleus where they phosphorylate various transcription factors. The proliferative effects of EGF, PDGF, VEGF and GFG are signaled through several pathways. The EGF, VEGF, FGF and PDGF receptors activates ras and the MAP kinase pathway, ultimately causing phosphorylation of transcription factors such as c-Fos to create AP-1 and ELK-1 that contribute to proliferation. Activation of STAT-1 and STAT-3 transcription factors by JAK kinases in response to growth factors contributes to proliferative signaling. Activated transcription factors (NFκB, STAT3 and others) then translocate into nucleus and transactivate many genes that regulate antioxidant defense, apoptosis, DNA damage repair, tissue remodeling and cell survival and thereby induce PC progression. In this perspective, this study clearly demonstrates that the seaweed polyphenols target every key player of this composition and may thus serve as effective ‘drug-deliverable’ for the mitigation of pancreatic carcinogenesis, cancer progression and dissemination to distant sites. Also, in an attempt to identify a fraction (or fractions) that may relatively have an upper hand in effecting anti-pancreatic response, we adopted inhibition weightage factor analysis across all polyphenol fractions for all measured parameters in all cell-lines investigated (Fig. 9B). This allows us to accumulate all endpoint data analyzed across all cell lines investigated and to draw more meaningful conclusions on identifying the potential benefit across the multifarious fractions of seaweed polyphenols examined. Interestingly, except for PT-DCM, the median values fall >5 for all seaweed fractions investigated. Notably, three fractions, SA-EA, PT-EA and HT-EA demonstrated a high upper quartile range of 9.0 (with >6.0 median *WF_inhibition_*) across the PC cells investigated.

In conclusion, for the first time this study identified the polarity dependent variations in total antioxidant capacity with maximal antioxidants in dichloromethane and ethyl acetate polyphenol fractions of brown sea weeds. Further this study clearly reveals that antioxidant rich sea weed polyphenols comprehensively promote anti pancreatic tumor potential, as evidently demonstrated by inhibitions of cell viability, proliferation and induced apoptotic cell death. Interestingly, cell proliferation studies utilizing normal human cells revealed the tumor-cell selective cell killing potential of these polyphenol fractions. In addition, this study has precisely shown the definite NFκB functional transcription-regulatory capacity of seaweed polyphenols. Interestingly, this study also demonstrates that seaweed polyphenols effectively target major key molecular players of PC progression. However, we clearly understand that it is too early to entertain any conclusions. As discussed above it is imminent to sequentially dissect out the active constituents from these fractions and characterize their antitumor capability and molecular orchestrations involved using appropriate animal models to derive a reasonable conclusion. The authors also recognize the limitations that it is practically impossible to follow the lead on every fractions and constituents. To that end, it is noteworthy; our team is also exploring the sulfated polysaccharides from the same source in PC setting. As differences in the extent of both potential and molecular signaling that we observed in the present study between established PC cells can be jettisoned by appropriate PC stem cell model, and are currently under investigation in our laboratory.

## Supporting Information

Figure S1 **Real time QPCR amplification charts for (A)** ***BCl2*** **, (B)** ***EGFR*** **, (C)** ***PDGFA*** **, (4)** ***VEGFA*** **, (5)** ***AKT*** **, (6)** ***hTERT*** **, (7)** ***kRAS*** **and (8)** ***FGFA*** **in in MiaPaCa-2, Panc-1, BxPC-3 and Panc-3.27 cells treated with 100 mg/ml of dichloromethane (DD-DCM, SA-DCM, SM-DCM, PT-DCM, HT-DCM) and ethyl acetate (DD-EA, SA-EA, SM-EA, PT-EA, HT-EA) seaweed polyphenol fractions.**
(TIF)

Table S1 **Weightage inhibition factor (** ***WF_inhibition_*** **) for molecular endpoints measured in pancreatic cancer (MiaPaCa-2, Panc-1, BxPC-3 and Panc-3.27) cell-lines exposed to dichloromethane (DD-DCM, SA-DCM, SM-DCM, PT-DCM, HT-DCM) and ethyl acetate (DD-EA, SA-EA, SM-EA, PT-EA, HT-EA) fractions.** Earmarking of weightage inhibition factor was based on the relative potential of each polyphenol fraction ranging from 1 to 10, 10 being highest cause effect potentiating fraction. These calculated *WF_inhibition_* values were utilized to plot box-and-whisker plot with immediate visuals of the center, the spread, and the overall range of distribution (Figure 9B).
(DOCX)
