# Supplementary figures and images for: Anti-Pancreatic Cancer Deliverables from Sea: First-Hand Evidence on the Efficacy, Molecular Targets and Mode of Action for Multifarious Polyphenols from Five Different Brown-Algae
Source: PLoS One. 2013 Apr 16;8(4):e61977. doi: 10.1371/journal.pone.0061977 (PMC3628576; doi:10.1371/journal.pone.0061977)

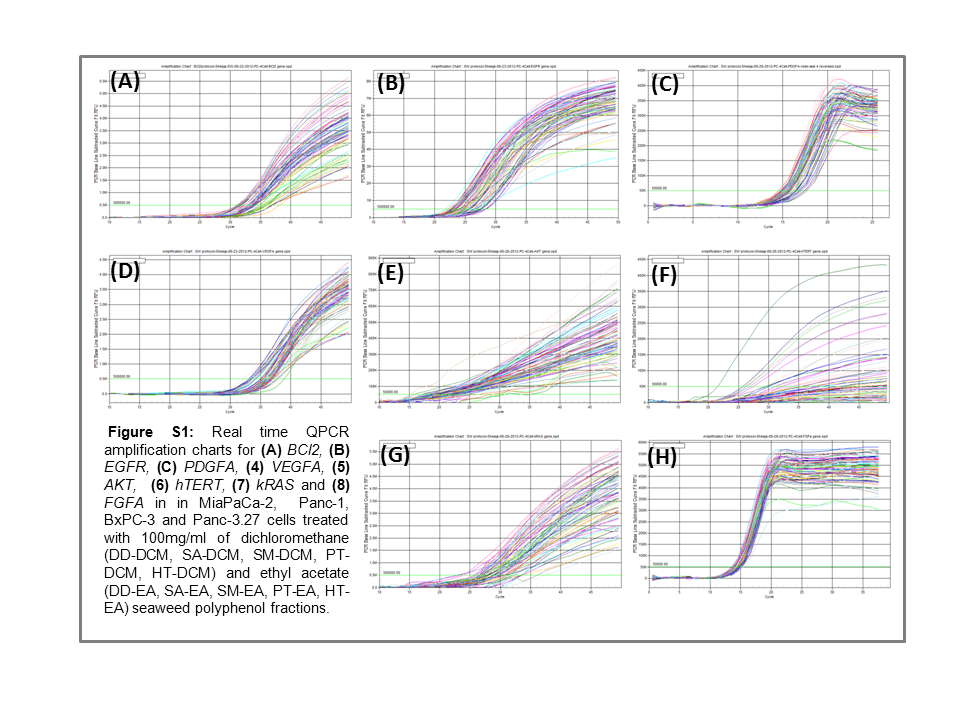

Supplement: Figure S1 — Real time QPCR amplification charts for (A) BCl2 , (B) EGFR , (C) PDGFA , (4) VEGFA , (5) AKT , (6) hTERT , (7) kRAS and (8) FGFA in in MiaPaCa-2, Panc-1, BxPC-3 and Panc-3.27 cells treated with 100 mg/ml of dichloromethane (DD-DCM, SA-DCM, SM-DCM, PT-DCM, HT-DCM) and ethyl acetate (DD-EA, SA-EA, SM-EA, PT-EA, HT-EA) seaweed polyphenol fractions. (TIF) [file pone.0061977.s001.tif]
