# Supplementary material for: Anti-Pancreatic Cancer Deliverables from Sea: First-Hand Evidence on the Efficacy, Molecular Targets and Mode of Action for Multifarious Polyphenols from Five Different Brown-Algae
Source: PLoS One. 2013 Apr 16;8(4):e61977. doi: 10.1371/journal.pone.0061977 (PMC3628576; doi:10.1371/journal.pone.0061977)
Supplement: Table S1 — Weightage inhibition factor ( WFinhibition ) for molecular endpoints measured in pancreatic cancer (MiaPaCa-2, Panc-1, BxPC-3 and Panc-3.27) cell-lines exposed to dichloromethane (DD-DCM, SA-DCM, SM-DCM, PT-DCM, HT-DCM) and ethyl acetate (DD-EA, SA-EA, SM-EA, PT-EA, HT-EA) fractions. Earmarking of weightage inhibition factor was based on the relative potential of each polyphenol fraction ranging from 1 to 10, 10 being highest cause effect potentiating fraction. These calculated WFinhibition values were utilized to plot box-and-whisker plot with immediate visuals of the center, the spread, and the overall range of distribution (Figure 9B). (DOCX) [file pone.0061977.s002.docx]

**Table S1:** Weightage inhibition factor (*WF_inhibition_*) for molecular endpoints measured in pancreatic cancer (MiaPaCa-2, Panc-1, BxPC-3 and Panc-3.27) cell-lines exposed to dichloromethane (DD-DCM, SA-DCM, SM-DCM, PT-DCM, HT-DCM) and ethyl acetate (DD-EA, SA-EA, SM-EA, PT-EA, HT-EA) fractions. Earmarking of weightage inhibition factor was based on the relative potential of each polyphenol fraction ranging from 1 to 10, 10 being highest cause effect potentiating fraction. These calculated *WF_inhibition_* values were utilized to plot box-and-whisker plot with immediate visuals of the center, the spread, and the overall range of distribution (Figure 9B).

| Molecular Endpoint | Methodology | Cell-line | DD-DCM | SA-DCM | SM-DCM | PT-DCM | HT-DCM | DD-EA | SA-EA | SM-EA | PT-EA | HT-EA |
| --- | --- | --- | --- | --- | --- | --- | --- | --- | --- | --- | --- | --- |
| BCL2-Transactivation | QPCR | MiaPaCa-2 | 3.0 | 6.0 | 8.0 | 10.0 | 9.0 | 5.0 | 1.0 | 4.0 | 7.0 | 2.0 |
| BCL2-Transactivation | QPCR | Panc-1 | 7.0 | 4.0 | 6.0 | 5.0 | 3.0 | 9.0 | 1.0 | 8.0 | 2.0 | 10.0 |
| BCL2-Transactivation | QPCR | BxPC-3 | 8.0 | 9.0 | 10.0 | 5.0 | 3.0 | 2.0 | 4.0 | 1.0 | 6.0 | 7.0 |
| BCL2-Transactivation | QPCR | Panc-3.27 | 3.0 | 4.0 | 5.0 | 6.0 | 2.0 | 1.0 | 7.0 | 8.0 | 9.0 | 10.0 |
| EGFR-Transactivation | QPCR | MiaPaCa-2 | 8.0 | 6.0 | 3.0 | 9.0 | 4.0 | 10.0 | 5.0 | 1.0 | 2.0 | 7.0 |
| EGFR-Transactivation | QPCR | Panc-1 | 6.0 | 5.0 | 4.0 | 1.0 | 9.0 | 3.0 | 10.0 | 7.0 | 2.0 | 8.0 |
| EGFR-Transactivation | QPCR | BxPC-3 | 1.0 | 10.0 | 3.0 | 5.0 | 6.0 | 9.0 | 7.0 | 8.0 | 4.0 | 2.0 |
| EGFR-Transactivation | QPCR | Panc-3.27 | 2.0 | 4.0 | 1.0 | 3.0 | 5.0 | 9.0 | 10.0 | 7.0 | 8.0 | 6.0 |
| PDGFA-Transactivation | QPCR | MiaPaCa-2 | 7.0 | 9.0 | 10.0 | 5.0 | 6.0 | 8.0 | 3.0 | 2.0 | 1.0 | 4.0 |
| PDGFA-Transactivation | QPCR | Panc-1 | 2.0 | 5.0 | 7.0 | 6.0 | 8.0 | 4.0 | 1.0 | 3.0 | 9.0 | 10.0 |
| PDGFA-Transactivation | QPCR | BxPC-3 | 9.0 | 7.0 | 3.0 | 2.0 | 10.0 | 4.0 | 1.0 | 6.0 | 8.0 | 5.0 |
| PDGFA-Transactivation | QPCR | Panc-3.27 | 9.0 | 5.0 | 9.0 | 10.0 | 1.0 | 4.0 | 7.0 | 3.0 | 2.0 | 6.0 |
| VEGF-Transactivation | QPCR | MiaPaCa-2 | 4.0 | 5.0 | 8.0 | 2.0 | 3.0 | 9.0 | 7.0 | 10.0 | 6.0 | 1.0 |
| VEGF-Transactivation | QPCR | Panc-1 | 1.0 | 7.0 | 3.0 | 6.0 | 4.0 | 2.0 | 9.0 | 5.0 | 8.0 | 10.0 |
| VEGF-Transactivation | QPCR | BxPC-3 | 10.0 | 4.0 | 3.0 | 6.0 | 9.0 | 2.0 | 1.0 | 7.0 | 5.0 | 8.0 |
| VEGF-Transactivation | QPCR | Panc-3.27 | 1.0 | 2.0 | 5.0 | 6.0 | 3.0 | 4.0 | 8.0 | 9.0 | 7.0 | 10.0 |
| AKT-Transactivation | QPCR | MiaPaCa-2 | 10.0 | 1.0 | 9.0 | 2.0 | 4.0 | 5.0 | 7.0 | 9.0 | 6.0 | 3.0 |
| AKT-Transactivation | QPCR | Panc-1 | 5.0 | 8.0 | 4.0 | 10.0 | 6.0 | 3.0 | 1.0 | 7.0 | 9.0 | 2.0 |
| AKT-Transactivation | QPCR | BxPC-3 | 9.0 | 7.0 | 3.0 | 4.0 | 5.0 | 8.0 | 2.0 | 1.0 | 10.0 | 6.0 |
| AKT-Transactivation | QPCR | Panc-3.27 | 8.0 | 10.0 | 3.0 | 4.0 | 1.0 | 9.0 | 2.0 | 6.0 | 5.0 | 7.0 |
| TERT-Transactivation | QPCR | MiaPaCa-2 | 8.0 | 4.0 | 1.0 | 8.0 | 10.0 | 6.0 | 10.0 | 2.0 | 3.0 | 5.0 |
| TERT-Transactivation | QPCR | Panc-1 | 3.0 | 5.0 | 8.0 | 10.0 | 10.0 | 7.0 | 4.0 | 1.0 | 2.0 | 6.0 |
| TERT-Transactivation | QPCR | BxPC-3 | 9.0 | 7.0 | 8.0 | 5.0 | 2.0 | 1.0 | 3.0 | 6.0 | 10.0 | 4.0 |
| TERT-Transactivation | QPCR | Panc-3.27 | 7.0 | 10.0 | 10.0 | 10.0 | 3.0 | 5.0 | 4.0 | 2.0 | 1.0 | 6.0 |
| kRAS-Transactivation | QPCR | MiaPaCa-2 | 7.0 | 3.0 | 2.0 | 6.0 | 8.0 | 5.0 | 10.0 | 4.0 | 1.0 | 9.0 |
| kRAS-Transactivation | QPCR | Panc-1 | 1.0 | 2.0 | 9.0 | 5.0 | 6.0 | 4.0 | 10.0 | 3.0 | 8.0 | 7.0 |
| kRAS-Transactivation | QPCR | BxPC-3 | 2.0 | 8.0 | 7.0 | 3.0 | 5.0 | 1.0 | 10.0 | 4.0 | 9.0 | 6.0 |
| kRAS-Transactivation | QPCR | Panc-3.27 | 2.0 | 8.0 | 7.0 | 3.0 | 5.0 | 1.0 | 10.0 | 4.0 | 9.0 | 6.0 |

**Supplementary Table 1 Contd.,**

| Molecular Endpoint | Methodology | Cell-line | DD-DCM | SA-DCM | SM-DCM | PT-DCM | HT-DCM | DD-EA | SA-EA | SM-EA | PT-EA | HT-EA |
| --- | --- | --- | --- | --- | --- | --- | --- | --- | --- | --- | --- | --- |
| FGF-Transactivation | QPCR | MiaPaCa-2 | 1.0 | 8.0 | 7.0 | 10.0 | 4.0 | 6.0 | 9.0 | 2.0 | 3.0 | 5.0 |
| FGF-Transactivation | QPCR | Panc-1 | 10.0 | 9.0 | 6.0 | 8.0 | 7.0 | 1.0 | 4.0 | 5.0 | 3.0 | 2.0 |
| FGF-Transactivation | QPCR | BxPC-3 | 7.0 | 5.0 | 4.0 | 2.0 | 10.0 | 6.0 | 3.0 | 8.0 | 9.0 | 1.0 |
| FGF-Transactivation | QPCR | Panc-3.27 | 4.0 | 3.0 | 2.0 | 8.0 | 1.0 | 5.0 | 6.0 | 7.0 | 10.0 | 9.0 |
| DNA damage | DNA Fragmentation | BxPC-3 | 1.0 | 3.0 | 5.0 | 4.0 | 9.0 | 8.0 | 10.0 | 2.0 | 7.0 | 6.0 |
| DNA damage | DNA Fragmentation | MiaPaCa-2 | 1.0 | 3.0 | 2.0 | 4.0 | 5.0 | 6.0 | 7.0 | 8.0 | 10.0 | 9.0 |
| DNA damage | DNA Fragmentation | Panc-1 | 1.0 | 4.0 | 6.0 | 2.0 | 8.0 | 10.0 | 9.0 | 7.0 | 3.0 | 5.0 |
| DNA damage | DNA Fragmentation | Panc-3.27 | 1.0 | 3.0 | 5.0 | 4.0 | 7.0 | 6.0 | 8.0 | 2.0 | 9.0 | 10.0 |
| pEGFR | Immunoblotting | Panc-1 |  | 8.0 | 4.0 | 2.0 | 3.0 | 6.0 | 5.0 | 9.0 | 7.0 | 10.0 |
| kRAS | Immunoblotting | Panc-1 |  | 6.0 | 2.0 | 5.0 | 3.0 | 4.0 | 7.0 | 8.0 | 9.0 | 10.0 |
| STAT3 | Immunoblotting | Panc-1 |  | 4.0 | 2.0 | 3.0 | 5.0 | 6.0 | 7.0 | 8.0 | 9.0 | 10.0 |
| AURkB | Immunoblotting | MiaPaCa-2 | 5.0 | 8.0 | 2.0 | 4.0 | 3.0 | 6.0 | 9.0 | 1.0 | 10.0 | 7.0 |
| kRAS | Immunoblotting | MiaPaCa-2 | 1.0 | 4.0 | 10.0 | 2.0 | 5.0 | 3.0 | 6.0 | 7.0 | 9.0 | 8.0 |
| STAT3 | Immunoblotting | MiaPaCa-2 | 6.0 | 2.0 | 1.0 | 3.0 | 7.0 | 5.0 | 9.0 | 4.0 | 10.0 | 8.0 |
| AURkB | Immunoblotting | BxPC-3 | 9.0 | 4.0 | 1.0 | 2.0 | 5.0 | 3.0 | 10.0 | 6.0 | 7.0 | 8.0 |
| kRAS | Immunoblotting | BxPC-3 | 1.0 | 4.0 | 2.0 | 3.0 | 8.0 | 5.0 | 10.0 | 9.0 | 7.0 | 6.0 |
| STAT3 | Immunoblotting | BxPC-3 | 1.0 | 3.0 | 2.0 | 4.0 | 7.0 | 5.0 | 9.0 | 8.0 | 6.0 | 10.0 |
| Cell Survival | Cell Proliferation | MiaPaCa-2 | 3.0 | 5.0 | 7.0 | 1.0 | 10.0 | 8.0 | 4.0 | 6.0 | 9.0 | 2.0 |
| Cell Survival | Cell Proliferation | Panc-1 | 8.0 | 4.0 | 2.0 | 10.0 | 9.0 | 7.0 | 3.0 | 1.0 | 5.0 | 6.0 |
| NFkB Transcription | Luciferase activity | Panc-1 | 5.0 | 2.0 | 9.0 | 10.0 | 3.0 | 8.0 | 7.0 | 1.0 | 6.0 | 4.0 |
| NFkB Transcription | Luciferase activity | BxPC-3 | 5.0 | 3.0 | 1.0 | 4.0 | 10.0 | 9.0 | 7.0 | 6.0 | 8.0 | 2.0 |
| NFkB Transcription | Luciferase activity | MiaPaCa-2 | 4.0 | 8.0 | 7.0 | 2.0 | 1.0 | 6.0 | 5.0 | 10.0 | 3.0 | 9.0 |
| Cell Survival | Cell Viability | MiaPaCa-2 | 2.0 | 1.0 | 5.0 | 4.0 | 10.0 | 7.0 | 6.0 | 8.0 | 9.0 | 3.0 |
| Cell Survival | Cell Viability | BxPC-3 | 9.0 | 5.0 | 8.0 | 6.0 | 9.0 | 5.0 | 7.0 | 4.0 | 10.0 | 3.0 |
| Cell Survival | Cell Viability | Panc-3.27 | 7.0 | 9.0 | 8.0 | 6.0 | 5.0 | 1.0 | 4.0 | 2.0 | 3.0 | 10.0 |
| Cell Survival | Cell Viability | Panc-1 | 2.0 | 8.0 | 10.0 | 1.0 | 5.0 | 9.0 | 4.0 | 7.0 | 3.0 | 6.0 |
